# How Useful Are Instrumental Examinations in Newly Diagnosed Children with ASD? Insights from Real-World Practice

**DOI:** 10.3390/children12070847

**Published:** 2025-06-27

**Authors:** Marilia Barbosa de Matos, Vittoria Vendrametto, Federica Molaschi, Federica Graziano, Martina Vacchetti, Barbara Svevi, Benedetto Vitiello, Daniele Marcotulli, Giuliana Delia, Franco Fioretto, Andrea Martinuzzi, Chiara Davico

**Affiliations:** 1 Section of Child and Adolescent Neuropsychiatry, Department of Public Health and Pediatric Sciences, University of Turin, 10125 Turin, Italy; marilia.barbosadematos@unito.it (M.B.d.M.); vittoria.vendrametto@unito.it (V.V.); federica.molaschi@unito.it (F.M.); benedetto.vitiello@unito.it (B.V.); daniele.marcotulli@unito.it (D.M.); chiara.davico@unito.it (C.D.); 2 Department of Psychology, University of Turin, 10125 Turin, Italy; 3 Section of Child and Adolescent Neuropsychiatry, Pediatric Hospital Regina Margherita, 10126 Turin, Italy; mvacchetti@cittadellasalute.to.it (M.V.); bsvevi@cittadellasalute.to.it (B.S.); 4 Department of Child and Adolescent Neuropsychiatry, A.S.L. CN1, 12084 Mondovì, Italyfranco.fioretto@aslcn1.it (F.F.)

**Keywords:** autism, ASD, EEG, MRI, instrumental examinations, genetic syndrome, comorbidity, diagnosis

## Abstract

Background: Autism spectrum disorder (ASD) is a neurodevelopmental condition with a complex and multifactorial etiology, diagnosed on the basis of clinical criteria. Medical comorbidities are common and often lead to instrumental examinations; however, the clinical utility of routinely performing such tests remains uncertain. This study aimed to assess the practical value of instrumental assessments in ASD by examining both prescribing behaviors and the prevalence of abnormal findings in a sample of autistic children. Methods: A combined-method approach was adopted: (1) an online survey of child neuropsychiatrists across the Piedmont region (Italy) explored current attitudes and practices regarding instrumental testing in children with ASD; (2) a retrospective cross-sectional analysis examined the frequency and clinical relevance of abnormal findings in ASD patients who underwent comprehensive testing at a tertiary hospital in Turin. Results: The survey showed that 85.7% of centers follow specific protocols for instrumental examinations, though practices vary considerably. Genetic testing and blood analyses are routinely performed, while EEG, MRI, audiometry, and metabolic screenings are generally based on clinical indication. In the retrospective study, instrumental tests revealed a low rate of clinically significant findings. Clinically relevant genetic abnormalities were detected in 7.9% of CGH-array tests. EEG abnormalities were seen in 9% of cases, though 57% had nonspecific or unclear results. Among biochemical parameters, notable findings included altered lipid profiles (45%), ferritin deficiency (24%), and anemia (12.5%) and no metabolic disorders were identified. Discussion: These findings highlight substantial variability in clinical practice and suggest that while some instrumental tests may provide valuable insights, routine screening is often of limited benefit. The high prevalence of nonspecific findings reinforces the need for careful clinical correlation, emphasizing the importance of balancing comprehensive assessment against the risks of over-testing and challenges in interpreting results. Future research should focus on developing evidence-based guidelines for instrumental assessments in this population.

## 1. Introduction

Autism spectrum disorder (ASD) is a neurodevelopmental condition characterized by social and communication impairments, alongside a tendency to engage in repetitive and/or restricted behaviors [1]. Its prevalence has steadily increased over recent years), with a current incidence of approximately 1 in 31 school-age children in the USA [2] and 1 in 75 in Italy [3].

Despite ongoing research, the etiology of ASD remains multifactorial and not fully understood. Diagnosis is clinical, as no validated biological markers or diagnostic laboratory tests are currently available [4]. However, some studies have offered some insights into the developmental alterations potentially resulting from gene-environment interactions [5].

Comorbidities and co-occurring conditions are highly prevalent in children with ASD and encompass a broad spectrum of neurodevelopmental, psychiatric, and medical conditions. These include intellectual disability, attention-deficit/hyperactivity disorder (ADHD), anxiety and mood disorders [6], as well as epilepsy, sleep disturbances, gastrointestinal issues, and syndromic or genetic disorders [7]. Accurate identification of these conditions is essential given their implications for treatment, prognosis, and genetic counseling.

Additional investigations such as genetic or metabolic testing, electroencephalography (EEG), and brain MRI may be necessary in selected cases, particularly when specific clinical indications are present. However, routine use of these assessments in the absence of red flags is not supported by strong evidence [4,8,9,10]. Furthermore, low compliance of children with ASD with medical procedures often complicates the decision-making process [11].

A recent systematic review and meta-analysis have highlighted a persistent lack of consensus among international guidelines regarding the type and extent of instrumental assessments recommended during the diagnostic evaluation of ASD. While hearing and vision assessments are broadly supported, the use of procedures such as EEG, MRI, or metabolic screening is generally recommended only in the presence of neurological or systemic abnormalities [12]. In contrast, a review of guidelines on genetic evaluation reported that some recommend chromosomal microarray (CMA) and Fragile X testing as first-tier assessments when no specific diagnosis is suspected. MECP2 testing is also recommended for females [13,14].

Moreover, actual clinical implementation of these recommendations remains inconsistent, influenced by physician practice patterns, healthcare policy frameworks, and available resources. In this context, the present study adopts an exploratory approach to investigate the use and clinical utility of instrumental examinations in real-world settings.

Focusing on newly diagnosed children with ASD in the Piedmont region of Italy, our study explores a central question: to what extent do instrumental investigations provide clinically relevant findings, and how well do current prescribing practices align with existing evidence and international guidelines? Specifically, the study aims to:

- describe local prescribing patterns for instrumental assessments in patients newly diagnosed with ASD;
- evaluate the prevalence and clinical relevance of abnormal findings;
- critically examine the potential benefits and limitations of instrumental testing in this population.

## 2. Materials and Methods

This study employs a combined methods approach:

- An online survey of child and adolescent neuropsychiatrists working in ASD-specialized centers to explore current practices in prescribing instrumental examinations for newly diagnosed children with ASD;
- A retrospective cross-sectional study of children with ASD who underwent instrumental assessments to determine the frequency of abnormal findings.

Together, these components aim to identify potential gaps between clinicians’ intended testing practices (as reported in the survey) and real-world diagnostic outcomes (from retrospective data), thereby informing evidence-based recommendations for ASD care protocols.

### 2.1. Online Survey

An online survey was conducted among various Child and Adolescent Psychiatry centers dedicated to ASD diagnosis within the public health system of the Piedmont region, Italy.

#### 2.1.1. Participants

Participants included child and adolescent neuropsychiatrists working in Local Health Facilities and Hospitals with dedicated ASD centers in the Italian public health system. These centers collectively serve an area with a population of 4,102,501 people, including 577,188 children aged 0–17 years [15].

#### 2.1.2. Data Collection

This survey-based study was conducted between September 2023 and January 2024 using an online questionnaire targeting child and adolescent neuropsychiatrists. The questionnaire was initially distributed via email using Google Forms, followed by two reminder emails sent at two-week intervals to non-respondents.

The online survey consisted of eight sections. The first section asked whether a protocol existed for instrumental assessments in patients with ASD. The remaining sections focused on specific types of examinations: genetic testing, blood chemistry, metabolic evaluation, EEG, MRI, audiometric, and ophthalmologic evaluations. Respondents were asked to indicate whether each type of examination was performed for all patients, some patients, or not at all. If not performed routinely, respondents were asked to specify common indications. They also estimated the percentage of abnormal findings observed in clinical practice. The full survey, including questions about protocol use and test-specific indications, is available in Supplementary Table S1.

### 2.2. Retrospective Cross-Sectional Study

Data were collected from the medical records of patients at the University of Turin Pediatric Hospital Regina Margherita, Torino, Italy, from February 2018 to April 2024.

#### 2.2.1. Participants and Procedures

This retrospective study reviewed medical records of children formally diagnosed with ASD according to [1,16] at the University of Turin—Pediatric Hospital Regina Margherita’s Outpatient and Day Hospital Service for Neurodevelopmental Disorders. The hospital is a tertiary care center with specialized outpatient and Day Hospital (DH) services for children with ASD.

Inclusion criteria were a formal ASD diagnosis made at our outpatient clinic and the availability of complete clinical and instrumental data. Patients diagnosed externally and referred solely for instrumental assessments from other centers were excluded due to incomplete medical records.

Following the clinical diagnosis, patients were referred to the DH service for further evaluation of possible comorbidities or genetic etiologies. This service is also accessible to children diagnosed at other regional centers, due to the DH team’s long-standing experience in managing ASD. Environmental adaptations were implemented to reduce sensory stimuli and improve compliance. All families admitted to the DH were offered a preparatory procedure, including a photo-based social story created using Augmentative and Alternative Communication (AAC) principles to help children understand the procedures. The routine exams included: (1) an EEG, (2) metabolic screening: amino acids in blood (AA blood); amino acids in urine (AA urine); very long-chain fatty acids (VCFLA); carnitine and acylcarnitine profile (CAO); (3) genetic screening: mutations for Fragile-X, PTEN (Phosphatase and Tensin Homolog), (for the ones with increased head circumference); (4) CMA; and (5) a blood test assessing hematological, nutritional and biochemical parameters.

Additional clinical and demographic variables were extracted from records: age at diagnosis, gender, bilingualism (yes/no), perinatal complications (yes/no), use of psychotropic medications, and BMI (normal, underweight, overweight). Neurodevelopmental and behavioral data included motor and language delays (yes/no), presence of functional communication (defined as the use of words, gestures, or AAC to express needs/emotions), food selectivity (yes/no), aggression/agitation (yes/no), and hyperactivity (yes/no).

#### 2.2.2. Statistical Analysis

Descriptive statistics were used to summarize the data: percentages for categorical variables and means with standard deviations for continuous variables. Analyses were performed using SPSS statistical software, version 29, for both study components. Differences between groups (with vs. without abnormal test findings) were analyzed using Chi-square tests for categorical variables and Mann–Whitney U tests for continuous variables that were not normally distributed.

Reference laboratory ranges for hematological, nutritional, and biochemical parameters included: Hemoglobin: 11.5–13.5 g/dL; Iron: 40–100 μg/dL; Ferritin: 22–275 μg/L; Vitamin B12: 300–950 ng/L; Folic acid: 3.0–16.0 μg/L; Glucose: 60–100 mg/dL; Total cholesterol: >200 mg/dL; Triglycerides: <105 mg/dL; HDL cholesterol: males < 35, females < 40 mg/dL; TSH: 0.810–7.020 mIU/L; Free thyroxine (fT4): 7.2–14.7 ng/dL

Effect sizes were calculated using Cramer’s V for Chi-square tests and the product-moment correlation coefficient (r) for nonparametric comparisons. A *p*-value of less than was considered statistically significant.

To summarize the overall estimated percentage of abnormal test results reported in the survey, which were reported by participants in predefined categories (<5%, 5–25%, 25–50%, 50–75%, and >75%), an estimated weighted mean was calculated.

#### 2.2.3. Ethical Considerations

This study complied with the ethical principles outlined in Resolution 196/96 and the Declaration of Helsinki (1975, revised in 2013). For the survey component, participant confidentiality and anonymity were maintained, and no financial incentives were provided. For the retrospective component, parental informed consent and ethics committee approval were obtained prior to data collection. All procedures followed international ethical standards and regulatory guidelines. The study was approved by the local Ethics Committee: Comitato Etico Territoriale “Interaziendale AOU Città della Salute e della Scienza di Torino”, Approval Code: 0097848, Approval Date: 24 September 2021.

## 3. Results

### 3.1. Survey

Of the 17 child and adolescent neuropsychiatric services in the region that were invited to participate, 14 completed the survey, each representing a distinct service. Among these, 12 centers (85.7%) reported having a protocol for conducting examinations in newly diagnosed ASD patients, while 2 centers (14.3%) did not. Evaluations were conducted in an inpatient setting at 35.7% of the centers and in an outpatient setting at 42.9%. A smaller proportion (14.3%) performed examinations either in an inpatient or outpatient setting, depending on the complexity of the required tests.

Genetic and blood chemistry tests were the most frequently prescribed, with 85.7% of centers performing them on all patients, while metabolic tests, EEG, MRI, audiometric, and ophthalmologic evaluations were more selectively used based on clinical indications. Common reasons for selective testing included neurological symptoms, developmental regression, dysmorphisms, and language or cognitive delays. The estimated percentage of abnormal results varied by test type, with genetic and EEG examinations most commonly yielding findings in the 5–25% range, and higher variability observed for metabolic, MRI, and blood chemistry assessments. Table 1 summarizes the findings.

### 3.2. Retrospective Cross-Sectional Study

#### 3.2.1. Patients’ Characteristics

The study population included 116 patients who met the inclusion criteria, selected from a total of 289 ASD patients evaluated between February 2018 and April 2024 at our ASD-dedicated day hospital service. As previously mentioned, patients who did not receive their ASD diagnosis at our hospital were excluded. Age at diagnosis ranged from 16 to 132 months, with a mean of 40.09 months (SD ± 19.76), and a male-to-female ratio of 3.6:1. DSM-5 severity levels were documented for 48 patients, with 22.9% classified as level 1, 41.7% as level 2, and 35.4% as level 3 support needs. Comorbidities were frequent (66.4%), including gastrointestinal issues (28.3%), epilepsy (8.9%), and sleep disorders (25.7%). Notably, 20.4% had two or more comorbidities. The main demographic and clinical characteristics are summarized in Table 2.

#### 3.2.2. Instrumental Examinations

The available exam results are reported in Table 3. Instrumental examinations revealed varying rates of abnormal findings across different modalities. Among genetic tests, 7.9% of CGH-array results were abnormal, and 15.8% showed findings of uncertain clinical significance. PTEN mutations were identified in 14.3% of the seven patients tested. Biochemical analyses showed elevated lipid levels in 45% of cases, low hemoglobin in 12.5%, iron deficiency in 12.3%, and low ferritin in 24%. Metabolic screenings did not yield any clinically significant results. The frequency of abnormal results also varied among other instrumental assessments. EEG abnormalities were found in 66% of patients, though only 9% were considered clearly clinically relevant. In contrast, audiometric evaluations and brain MRIs were largely normal. Table 3 summarizes the findings.

#### 3.2.3. Association Between Abnormal Exams and Clinical Variables

Associations between normal and abnormal examination results were analyzed for clinical variables, including gender, perinatal complications, DSM severity levels, motor delay, presence of comorbidities, food selectivity, language delay, functional communication, aggressive behavior, hyperactivity, use of psychotropic medication, and BMI abnormalities. There was a negative association between iron deficiency and food selectivity (*p* = 0.043; Cramer’s V = 0.24), a positive association between ferritin deficiency and language delay (*p* = 0.033; Cramer’s V = 0.23), and between anemia (low hemoglobin) and motor delay (*p* = 0.026; Cramer’s V = 0.21), as well as between aggressive behavior and dyslipidemia (*p* = 0.025; Cramer’s V = 0.24). Nonetheless, effect size values (Cramer’s V ≤0.2) indicate weak associations between these variables. No other statistically significant associations were found.

#### 3.2.4. Comparison Between Clinicians’ Estimate and Real World Findings

A descriptive comparison of the online survey results and the retrospective cross-sectional study is presented in Table 4. There is a strong concordance between clinicians’ expectations and actual findings for genetic testing, blood chemistry tests, metabolic screenings, and MRI, particularly when considering the proportion of clinically significant abnormalities. However, a notable discrepancy emerged with EEG findings: clinicians expected 18.8% abnormal results, whereas the study observed a much higher rate of 66%, although only 9% were clearly clinically significant. Conversely, audiometric abnormalities were overestimated in the survey, with an expected 11.7% compared to only 1.2% abnormal results observed and none clinically significant. Ophthalmologic evaluations were not performed in the retrospective study.

## 4. Discussion

This study investigated the routine use and potential clinical relevance of instrumental examinations in children newly diagnosed with ASD. This was achieved through two approaches: a survey of child and adolescent neuropsychiatrists in specialized centers within the Piedmont region of Italy, and a retrospective cross-sectional study of children with ASD who had undergone extensive instrumental examination.

The survey revealed that the majority (85.7%) of centers reported having protocols for instrumental examinations for these children, but with significant heterogeneity. Among the centers, there was strong consensus on universal genetic testing and blood chemistry tests, which were performed by 85.7% of them. In contrast, universal prescription was less common for MRI (21.4%), EEG (34.7%), metabolic tests (35.7%), and audiometric evaluations (28.6%), which were predominantly reserved for cases presenting specific clinical red flags. Ophthalmologic evaluations show the greatest disparity in practice: while performed universally by half the respondents, they were not performed at all by the remaining 42.9%.

These findings reflect the variability and lack of consensus highlighted in recent literature: while routine use of EEG, MRI, and metabolic testing is generally discouraged without specific clinical indications, hearing and vision assessments are widely supported. Some guidelines also recommend genetic evaluations, particularly CMA and Fragile X testing [4,8,9,10,12].

Hereafter, each test is discussed separately, combining survey and retrospective data results, together with reflections on the potential benefits and limitations of instrumental testing in children with ASD.

### 4.1. Genetic Testing

Our retrospective study revealed a slightly lower proportion of abnormal genetic findings (12.6%) compared to the survey data (18.5%). The prevalence of abnormalities involving a known pathogenic mutation was only 4.2%. This aligns with previous studies reporting varying prevalence rates of genetic abnormalities in individuals with ASD ranging from approximately 11.2% to 17.5% [17,18].

Current guidelines, including those from the American College of Medical Genetics and Genomics and the International Standards for Cytogenomic Arrays Consortium, recommend genetic testing such as CMA and Fragile X DNA analysis for children with ASD [14]. In our sample, no clear associations were found between CMA results and specific clinical variables. However, identifying an underlying genetic syndrome may still have significant clinical value. Even if it does not directly influence the management of core ASD symptoms, a genetic diagnosis can inform the management of associated medical and psychiatric comorbidities, prognosis, recurrence risk, and therapeutic strategies [19]. In addition, genetic information can be of value to families planning future pregnancies.

Thus, genetic testing in ASD offers both potential benefits and significant limitations. While routine screening has limited clinical utility, a targeted approach guided by clinical presentation and family history can help identify actionable genetic conditions. This is particularly relevant in syndromic forms of ASD, where genetic findings may clarify the etiology and inform medical management [20]. However, many test results are normal or unclear, which can confuse or disappoint families expecting clear answers [21]. Uncertain findings may also cause stress for parents already dealing with an ASD diagnosis [20]. Therefore, clinicians should carefully decide when to offer genetic testing and provide clear counseling to help families understand the possible outcomes and challenges.

### 4.2. Blood Chemistry Tests

Blood chemistry testing showed close alignment between our retrospective findings and the survey responses from neuropsychiatrists. While 16.5% of patients in our study had abnormal results, 14.8% were considered clinically significant, closely matching the 13.4% reported in the survey. This suggests a more uniform understanding and application of these assessments across clinical settings.

In our sample, a significant negative association was observed between iron deficiency and food selectivity (*p* = 0.043), this unexpected result might be explained by the hypothesis that children with restrictive eating patterns, who are typically at higher risk for nutritional deficiencies [22], may have already received targeted dietary interventions or supplementation. Additional findings included an association between ferritin deficiency and both language delay (*p* = 0.019) and motor delay due to anemia (*p* = 0.026). These results highlight the potential impact of iron deficiency on neurodevelopment. Existing literature supports the role of iron in cognitive, behavioral, and motor development [23,24]. A novel observation in our study was the positive association between aggressive behavior and dyslipidemia (*p* = 0.015), suggesting a potential link between metabolic status and behavioral challenges in ASD. This aligns with evidence from other populations showing that lipid imbalances can affect mood and behavior [25]. These findings point to meaningful connections between nutritional status, development, and behavior in ASD. However, given the cross-sectional design of our study, causal inferences cannot be drawn.

Nutritional assessments, including vitamin and mineral levels, may offer important clinical benefits for children with ASD, particularly those with gastrointestinal disorders, food selectivity, or developmental delays, all of which are highly prevalent in this population [26]. Many nutritional deficiencies are treatable, yet often remain undetected due to their subtle or oligosymptomatic presentation. In this context, routine blood screening can be a valuable tool for early identification of underlying medical conditions and nutritional deficits that may otherwise be missed, potentially informing timely interventions.

However, these potential benefits must be critically weighed against important limitations. Performing blood tests in children with ASD often presents practical challenges. Many of these children experience heightened sensitivity to sensory stimuli and may find medical procedures particularly distressing. The use of coercive measures to obtain samples can result in lasting psychological harm and increase the burden of care for families and caregivers [27]. Thus, while blood chemistry testing holds diagnostic and preventive value, its implementation should be guided by clinical necessity and individualized assessment, taking into account both potential yield and the invasiveness of the procedure.

### 4.3. Metabolic Assessment

Metabolic testing revealed a strong concordance between clinicians’ expectations and the actual diagnostic yield. In the survey, clinicians estimated that fewer than 5% of cases would show abnormal results. This expectation was closely mirrored by the retrospective study, where only 1.2% of metabolic tests showed any abnormalities, none of which were clinically significant.

Individual inborn errors of metabolism (IEMs) are rare, but together they occur more often, affecting about 1 in every 800 to 3000 live births [28,29]. The most common IEMs reported in children with ASD are mitochondrial dysfunction and cerebral folate abnormalities [30]. Usually, IEMs linked to ASD cause multiple symptoms like lethargy, seizures, vomiting, unusual physical features, or developmental regression. Therefore, ASD alone is rarely the first sign of a metabolic disorder [7]. Several studies [31,32,33] have found no support for routine metabolic testing in children with non-syndromic ASD. Instead, testing should be done only when specific clinical signs suggest a metabolic problem [34].

Although metabolic disorders are uncommon, some can be treated, and early diagnosis can greatly improve outcomes. In Italy, newborn screening tests for about 45 metabolic diseases, including phenylketonuria, maple syrup urine disease (MSUD), homocystinuria, galactosemia, and tyrosinemia types I–III, allowing early treatment [35]. However, some treatable IEMs, such as certain lysosomal storage disorders (like Mucopolysaccharidoses types I, II, and III), creatine deficiency syndromes, and X-linked adrenoleukodystrophy, are not part of routine screening [36].

Therefore, while instrumental metabolic testing can be highly beneficial in patients showing clinical red flags suggestive of metabolic disease, its routine use in unselected ASD populations is limited by a low diagnostic yield and lack of supporting evidence. This highlights the importance of targeted, clinically driven testing rather than broad metabolic screening in all children with ASD.

### 4.4. EEG

Neuropsychiatrists in the survey estimated an EEG abnormality rate of approximately 18.8%, while our retrospective study found a much higher rate of 66%. This difference likely reflects the high prevalence of nonspecific or unclear EEG abnormalities in children with ASD. In fact, 57% of the abnormal results lacked clinical significance, highlighting the challenges in interpreting EEG findings in this population and emphasizing the need for careful clinical correlation.

EEG abnormalities are common in ASD, with documentation in 23% to 80% of non-epileptic children [36], but their clinical significance, particularly in the absence of seizures, remains uncertain [37]. While several studies have explored these abnormalities as potential biomarkers of altered neural connectivity in ASD [38], our study found no significant associations between EEG findings and clinical variables, supporting previous literature that suggests routine EEG screening may not be necessary in children with ASD who do not exhibit clinical signs of epilepsy [39].

Clinicians should be cautious about the limitations of performing unnecessary EEGs, as they can be difficult to interpret, stressful, and costly for families. Furthermore, many EEG abnormalities lack clear clinical significance, which limits their practical value. Despite these limitations, EEG remains useful for identifying specific conditions such as Landau-Kleffner Syndrome and continuous spikes and waves during sleep (CSWS), both characterized by language regression associated with continuous epileptic activity during sleep [40]. Therefore, while routine EEG screening has limited benefit in unselected ASD populations, it retains important diagnostic value in targeted clinical situations.

### 4.5. MRI

In the survey, abnormal MRI findings were reported in 13.7% of cases, while our cross-sectional study identified a slightly higher rate of 20%. A previous study reported even higher rates, with 37.4% incidental and 27.1% pathological MRI findings, of which 18.3% were considered definite pathology [41]. These discrepancies may result from differing definitions of abnormality and variations in the populations studied.

Advances in neuroimaging have increasingly contributed to understanding the neural mechanisms of ASD, revealing both anatomical and metabolic abnormalities that may aid research into the disorder’s etiologies and comorbidities [42,43]. However, due to ASD’s heterogeneity and difficulties in generalizing diagnostic findings, many neuroimaging abnormalities lack clear clinical significance. Consequently, neuroimaging is not routinely recommended for ASD diagnosis [41].

There are several limitations to the use of MRI in the assessment of individuals with ASD. Its diagnostic utility is generally limited in this population. Additionally, the procedure is costly, often requires sedation, and carries a significant risk of overinterpreting incidental findings that may lack clinical relevance. These factors highlight the importance of reserving MRI for cases with specific indications, such as atypical clinical features, developmental regression, or coexisting neurological conditions. In such cases, MRI can provide valuable insights to guide diagnosis and management. However, clinicians must carefully weigh the potential benefits against the inherent risks and burdens of the procedure, ensuring clear and transparent communication with families throughout the decision-making process [41].

### 4.6. Hearing Assessment

In our cross-sectional study, audiometric evaluation was performed in 50% of the patients, with abnormalities detected in only one case. Notably, the survey estimated a rate of abnormal audiometric findings of 11.7%, suggesting that clinicians consider hearing impairments to be relatively uncommon but not rare in this population.

A systematic review suggests that brainstem pathology is often present in individuals with ASD, which aligns with commonly observed auditory abnormalities such as atypical sound sensitivity, poor sound localization, and difficulty filtering background noise [44]. These findings indicate that auditory evaluations might have a role in assessing risk for ASD [45].

Hearing plays a crucial role in sensory processing and effective oral communication, suggesting that comprehensive hearing assessments could be beneficial in evaluating children with ASD. Although some guidelines recommend hearing evaluations for newly diagnosed children [12], there is limited evidence to support routine auditory screening for all children within this population. Auditory impairments can mimic or worsen ASD symptoms, underscoring the clinical relevance of hearing assessments; however, universal screening may not be the most cost-effective or feasible strategy. Instead, a targeted approach focusing on children with specific risk factors, such as language delays, suspected hearing loss, family history of hearing impairment, or recurrent ear infections, may better optimize resource use while enhancing clinical outcomes.

### 4.7. Visual Assessment

Interestingly, 42.9% of centers did not request ophthalmologic evaluations, possibly due to the challenges involved in assessing vision in children with ASD. However, several studies have reported higher rates of visual problems in children with ASD compared to age-matched peers, with even greater prevalence among those with comorbid intellectual disability [46]. Moreover, children with severe visual impairment often exhibit more autism-like behaviors than their non-visually impaired counterparts [47], which can lead to misdiagnosis or overlooked vision issues.

The American Academy of Pediatrics (AAP) recommends that visual screening be attempted between 12 months and 3 years of age for all children [48]. Similarly, the American Academy of Ophthalmology (AAO), the American Association for Pediatric Ophthalmology and Strabismus (AAPOS) uniformly recommend annual visual acuity screening from ages 3 to 5 to detect conditions such as amblyopia or its risk factors, including strabismus, anisometropia, and refractive errors [48].

For younger, preverbal, or developmentally delayed children with ASD, vision screening often requires specialized instruments to accommodate their unique challenges. While routine screenings can help detect common ophthalmic conditions, the need for specialized assessment tools and the difficulties in cooperation may limit the feasibility and accuracy of testing in this population. Children who present with specific ophthalmic signs or are at high risk for eye diseases should be promptly referred to specialists to ensure timely diagnosis and intervention. Screening should continue every other year from age 6 onward to monitor and manage emerging visual issues [48]. Given these considerations, incorporating vision care as a routine component of the comprehensive assessment for children with ASD can offer significant benefits by identifying treatable visual impairments, although clinicians must also be mindful of the practical limitations and tailor testing approaches accordingly.

### 4.8. Limitations

This study has several limitations. The survey data rely on self-report and may be subject to recall bias. Moreover, although included centers serve a huge proportion of Piedmont territory, such area may not be representative of the whole country. The retrospective chart review is limited by the availability and quality of data in medical records. Our sample size may have limited our power to detect statistically significant associations. Finally, our findings may not be generalizable to other populations or healthcare settings. The sample may be subject to selection bias, as it includes tertiary care centers where more complex and severe cases are likely to be treated. This study has several important limitations. First, both components—the survey and the retrospective analysis—are geographically restricted to the Piedmont region of Italy. While this offers a detailed view of local clinical practices, it limits the generalizability of the findings to other regions or healthcare systems with different diagnostic protocols, resources, and socio-economic conditions.

Second, the retrospective cross-sectional analysis was conducted at a tertiary referral center, which may introduce selection bias, as more complex or severe cases are likely to be overrepresented. This could affect the observed rates of comorbidities and abnormal test findings. Moreover, the retrospective design limits the ability to draw causal inferences.

Third, although the sample size is sufficient for descriptive analyses, it may be underpowered to detect less common or more subtle associations. Additionally, the findings may not reflect practices in centers that did not participate in the survey or in patients assessed outside the included institutions.

Finally, the survey data are based on self-report, which introduces potential recall bias and variability in how respondents interpret diagnostic yield and testing practices.

Despite these limitations, the study offers meaningful real-world insights into current instrumental examination practices and outcomes. These findings may support a more selective and evidence-based approach to the use of instrumental assessments in the diagnostic workup of children with ASD.

## 5. Conclusions

This study provides a comprehensive overview of instrumental assessment practices in children with ASD in the Piedmont region of Italy. A key strength is its real-world design, combining prescriber perspectives with actual clinical data, offering practical insights that are directly applicable to refining diagnostic protocols for ASD.

Our findings reveal considerable variability in the use of instrumental examinations for newly diagnosed children with ASD. The relatively low percentage of abnormal findings in many tests raises questions about their routine application, suggesting potential over-testing in some cases—even though clinical benefit is not exclusively linked to abnormal results.

It is important to recognize that the clinical utility of an instrumental examination is not solely dependent on detecting abnormalities. Normal results can offer significant clinical value by conclusively excluding suspected pathologies or differential diagnoses, thereby providing reassurance to both patients and their families.

The selection of examinations should primarily be guided by clinical suspicions derived from a thorough anamnesis and physical examination, ensuring alignment with established evidence-based guidelines and practice recommendations. However, prescribing habits in real-world settings are influenced by several other factors, including parental expectations for active intervention. Clinicians must remain mindful of the risk of colluding with parental anxiety, which may lead to unnecessary testing. Additionally, the clinician’s individual experience and training, the widespread practice of defensive medicine driven by medico-legal concerns, the availability of advanced diagnostic technologies, and institutional protocols or local clinical customs all play significant roles in decision-making.

Furthermore, deciding whether to perform an examination involves weighing additional critical considerations. The possibility of incidental findings poses a risk of over-treatment, complicating clinical decisions. Economic costs also warrant careful evaluation, particularly within public health systems such as Italy’s. Finally, a thorough risk-benefit analysis is essential—especially for procedures requiring sedation.

While these findings offer valuable insights into current practices, it is essential to consider the long-term implications of instrumental examinations. Future research should focus on prospective studies assessing the long-term utility of early instrumental findings, multicenter evaluations of guideline implementation, and cost-benefit analyses to inform evidence-based policy decisions. Additionally, each center should critically assess the clinical relevance of these examinations in light of their specific patient populations and available economic resources.

## Figures and Tables

**Table 1:** Online survey of child and adolescent neuropsychiatric on attitudes towards instrumental examinations prescriptions in newly diagnosed children with ASD.

Examination Type	Performed on All (%)	Performed on Some (%)	Not Performed (%)	Common Indications	Estimated % Abnormal Results
Genetic Testing	85.7	14.3	0	Family history Dysmorphisms	5–25% (87.7%) 25–50% (14.3%)
Blood Chemistry Tests	85.7	14.3	0	History of hypotonia Pharmacological treatment Intellectual disability	None (7.1%) <5% (42.9%) 5–25% (28.6%) 25–50% (21.4%)
Metabolic Tests	35.7	50	14.3	Neurological symptom Suspected metabolic disease Developmental regression Encephalopathy Family history	None (41.7%) <5% (58.3%)
EEG	35.7	64.3	0	Seizures Family history of epilepsy Neurological symptoms Language regression Encephalopathy Developmental delay Paroxysmal phenomena	<5% (28.6%) 5–25% (57.1%) 50–75% (15.3%)
MRI	21.4	71.4	0	Dysmorphisms neurologic signs and symptoms cognitive delay severe language delay encephalopathy regression epilepsy	<5% (50%) 5–25% (41.9%) 75–100% (7.1%)
Audiometric Evaluations	28.6	57.1	0	Language impairment Suspected hearing loss Family history of hearing loss Frequent ear infections	None (15.4%) <5% (7.7%) 5–25% (76.9%)
Ophthalmologic Evaluations	50	7.1	42.9	Suspected visual impairment Deficits in eye movement Abnormal eye screening at 3 years Evaluation of gaze praxis	None (20%) <5% (50%) 5–25% (30%)

**Table 2:** Demographics and clinical characteristics of the sample (*n* = 116).

Variable (*n*)	*n* (%)
Gender (116 *)	
Female	25 (21.6)
Male	91 (78.4)
DSM-5-TR severity (48 *)	
Level 1	11 (22.9%)
Level 2	20 (41.7%)
Level 3	17 (35.4%)
Bilingualism (116 *)	36 (31%)
Perinatal complications (116 *)	58 (50%)
Neurodevelopmental/ Behavioral	
Motor Development Delay (114 *)	72 (63.2%)
Speech Delay (112 *)	108 (96.4%)
Language Regression (112 *)	2 (1.8%)
Functional Communication Skills (111 *)	61 (52.6%)
Irritability/Aggression (110 *)	41 (37.3%)
Hyperactivity (108 *)	34 (31.5%)
Food Selectivity (116 *)	56 (48.3%)
Comorbidities (113 *)	75 (66.4%)
Gastrointestinal Issues	32 (28.3%)
Epilepsy	10 (8.9%)
Recurrent Infection	6 (5.3%)
ADHD	5 (4.4%)
ARFID	2 (1.8%)
Tic/Tourette	3 (2.7%)
Sleep Disorders	29 (25.7%)
Delayed sleep-wake phase/insomnia disorder	13 (11.5%)
Sleep apnea	1 (0.9%)
Sleep-related movement disorders/parasomnia	2 (1.8%)
Two or more comorbidities (113 *)	23 (20.4%)
BMI (93 *)	
Overweight	21 (22.6%)
Underweight	17 (18.3%)
Use of medication (113 *)	13 (11.5%)
Two or more medications (113*)	5 (4.3%)

* Percentage based only on valid cases.

**Table 3:** Instrumental examinations (*n* = 116).

Exam (*n*)	Abnormal *n* (%)	Abnormal: Clinically Irrelevant or Unclear *n* (%)	Normal *n* (%)
Genetic tests			
Fragile X (106 *)	0	2 (1.9%)	104 (98.1%)
CGH-array (101 *)	8 (7.9%)	16 (15.8%)	77 (76.2%)
PTEN (7 *)	1 (14.3%)	0	6 (85.7%)
Biochemical			
TSH (100 *)	0	13 (11.8%)	97 (88.2%)
Hemoglobin (112 *)	14 (12.5%)	0	98 (87.5%)
Glucose: (110 *)	6 (5.5%)	0	104 (94.5%)
Lipids (109 *)	49 (45%)	0	60 (55%)
B12 (105 *)	3 (2.6%)	0	102 (97.1%)
Iron (106 *)	13 (12.3%)	0	93 (87.8%)
Ferritin (104 *)	25 (24%)	0	79 (76%)
Metabolic screening			
Plasma AA (97 *)	0	1 (1%)	96 (99%)
Urinary AA (94 *)	0	2 (2.1%)	92 (97.9%)
VCFLA (62 *)	0	0	62 (100%)
CAO (80 *)	0	1 (1.2%)	79 (98.8%)
EEG (100 *)	9 (9%)	57 (57%)	34 (34%)
MRI (20 *)	4 (20%)	0	16 (80%)
Audiometry (53 *)	1 (1.2%)	0	52 (98.12%)

* Percentage based only on valid cases.

**Table 4:** Comparison of Abnormal Test Results *(Survey* vs. *Cross-Sectional Study)*.

Test	Estimated Weighted Mean (%)	Cross-Sectional Study: Abnormal Results (%)/Clinically Relevant (%)
Audiometry	11.7	1.2/0
Ophthalmologist	5.75	not performed
Genetics	18.5	12.6/4.2
Blood Chemistry Tests	13.4	16.5/14.8
Metabolic Screening	1.46	1.2/0
EEG	18.8	66/9
MRI	13.7	20/0

## Data Availability

The data presented in this study are available upon reasonable request from the corresponding author. The data are not publicly available due to privacy and ethical considerations.

## Supplementary Materials

The following supporting information can be downloaded at: https://www.mdpi.com/article/10.3390/children12070847/s1, Table S1: Survey on examination practices.
