# Supplementary material for: How Useful Are Instrumental Examinations in Newly Diagnosed Children with ASD? Insights from Real-World Practice
Source: Children (Basel). 2025 Jun 27;12(7):847. doi: 10.3390/children12070847 (PMC12293674; doi:10.3390/children12070847)
Supplement: Supplementary file 1 [file children-12-00847-s001.zip › children-3683487-supplementary.pdf]

Supplementary Material

Table S1. Survey on examination practices.

Category	Question	Response Options
**General Protocol**	Is there an examination protocol for autism patients?	Yes / No
**Examination Setting**	What is the examination setting?	Inpatient setting/ outpatient setting/ Other
	% of abnormal results:	None / <5% / 5-25% / 25-50% / 50-75% / >75%
**Genetic Tests**	Are genetic tests conducted?	All patients / Some patients / Not at all
	Indication (if conducted for some patients):	Open answer
	% of abnormal results:	None / <5% / 5-25% / 25-50% / 50-75% / >75%
**Blood Tests**	Are blood tests conducted?	All patients / Some patients / Not at all
	Indication (if conducted for some patients):	Open answer
	% of abnormal results:	None / <5% / 5-25% / 25-50% / 50-75% / >75%
**Metabolic Tests**	Are metabolic tests conducted?	All patients / Some patients / Not at all
	Indication (if conducted for some patients):	Open answer
	% of abnormal results:	None / <5% / 5-25% / 25-50% / 50-75% / >75%
**EEG**	Is EEG conducted?	All patients / Some patients / Not at all
	Indication (if conducted for some patients):	Open answer
	% of abnormal results::	None / <5% / 5-25% / 25-50% / 50-75% / >75%
**Brain MRI**	Is brain MRI conducted?	All patients / Some patients / Not at all
	Indication (if conducted for some patients):	Open answer
	% of abnormal results:	None / <5% / 5-25% / 25-50% / 50-75% / >75%
**Audiometric Evaluation**	Is audiometric evaluation conducted?	All patients / Some patients / Not at all
	Indication (if conducted for some patients):	Open answer
	% of abnormal results:	None / <5% / 5-25% / 25-50% / 50-75% / >75%
**Ophthalmological Evaluation**	Is ophthalmological evaluation conducted?	All patients / Some patients / Not at all
	Indication (if conducted for some patients):	Open answer
	% of abnormal results:	None / <5% / 5-25% / 25-50% / 50-75% / >75%
